# P17 Baseline characteristics, disease progression rates and risk factors among female patients with uncomplicated urinary tract infection in England

**DOI:** 10.1093/jacamr/dlad077.021

**Published:** 2023-08-02

**Authors:** Mark Wilcox, Dave Heaton, Aruni Mulgirigama, Ashish V Joshi, Viktor Chirikov, Daniel C Gibbons, David Webb, Xiaocong L Marston, Myriam Alexander, Fanny S Mitrani-Gold

**Affiliations:** University of Leeds & Leeds Teaching Hospitals, Leeds, UK; OPEN Health, Marlow, UK; GSK, Brentford, London, UK; GSK, Collegeville, PA, USA; OPEN Health, Bethesda, MD, USA; GSK, Brentford, London, UK; GSK, Brentford, London, UK; OPEN Health, Marlow, UK; OPEN Health, Marlow, UK; GSK, Collegeville, PA, USA

## Abstract

**Background and Objectives:**

Uncomplicated urinary tract infection (uUTI) is a common, usually community-acquired bacterial infection. Little is known about risk factors for disease progression (defined here as hospitalization for *Escherichia coli* sepsis, bacteraemia or acute pyelonephritis) among patients with uUTI. We report the baseline demographic and clinical characteristics among female uUTI patients with/without disease progression in England.

**Methods:**

This retrospective cohort study utilized anonymized patient data from 1 January 2018 to 31 December 2019 from the Clinical Practice Research Datalink (CPRD) database linked to English Hospital Episode Statistics data. Eligible patients were female, aged ≥12 years, had received an index diagnosis for a community-acquired uUTI, had ≥12 months’ CPRD data history, and had received ≥1 oral antibiotic within ±5 days of diagnosis. Patients were excluded if they had complicated UTI or complicating comorbidities, were hospitalized/visited an accident and emergency department 28 days prior to index or had received IV antibiotics as initial therapy. uUTI episodes were defined as the 28 days post-index (Figure 1). Descriptive patient (demographic and clinical characteristics), region and practice-level characteristics in the 12 month baseline period are presented for patients with/without disease progression.

**Figure 1.**
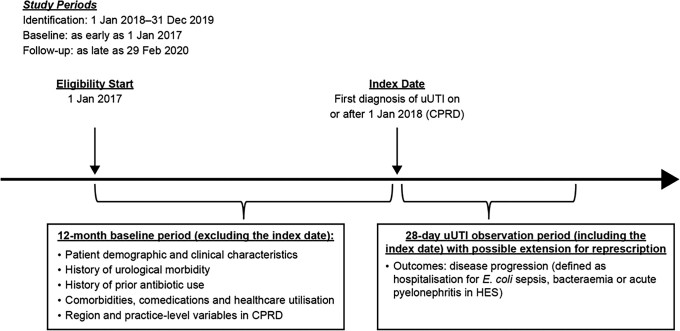
Study design. HES, Hospital Episode Statistics.

**Results:**

Among 116793 female patients with a uUTI, 207 were hospitalized for *E. coli* sepsis, bacteraemia or acute pyelonephritis within 28 days of index (0.2%). The mean age (SD) for patients with/without progression was 67.3 (20.2) and 52.9 (21.1), respectively; 81% and 34% of patients with/without progression were ≥50 years old. Imbalances were observed for comorbid burden, baseline medications, initial presentation (i.e. home visit requirement) and prior antimicrobial treatment (Table 1). Few regional or practice-level differences were apparent (Table 1).

**Table 1.** Patient (demographic and clinical characteristics), region and practice-level variable distribution between uUTI patients without and with hospitalization for uUTI disease progression

Variable description/Statistic or category	Patient subgroups			
	no disease progression (*N*=116586)		disease progression (*N*=207)	
	*N*	%	*N*	%
Patient-level				
Age at diagnosis, years, mean (SD)	52.9 (21.1)	–	67.3 (20.2)	–
Age at diagnosis				
≥12 to <16	711	0.6	0	0
≥16 to <30	19861	17	15	7.2
≥30 to <40	16682	14	13	6.3
≥40 to <50	15184	13	11	5.3
≥50 to <60	24512	21	42	20
≥65	39636	34	126	61
Age at diagnosis (pooled)				
<50	52438	45	39	19
≥50	39636	34	168	81
Charlson comorbidity index, mean (IQR)	0 (0, 0)	–	0 (0, 1)	–
Charlson comorbidity category				
0	95944	82	134	65
1	18007	15	58	28
2	2300	2	12	5.8
≥3	335	0.3	<5	
Race/ethnicity, N (%)				
Asian or Asian British	5161	4.4	6	2.9
Black or Black British	3287	2.8	6	2.9
Chinese or other group	1995	1.7	<5	-
Ethnic group not recorded	22781	20	43	21
White	41340	35	73	35
Number of hospital admissions in prior year, mean (IQR)	0 (0, 0)	–	0 (0, 0)	–
Number of A&E attendances in prior year, mean (IQR)	0 (0, 1)	–	0 (0, 1)	–
Smoking status				
Non-smoker	72546	62	119	57
Ex-smoker	27084	23	62	30
Smoker	16956	15	26	13
Menopausal status, yes	34072	29	60	29
Obesity, yes	13858	12	33	16
Mild/moderate renal impairment, yes	2262	1.9	10	4.8
Controlled diabetes (HgA1C <6.5/7%), yes	2451	2.1	14	6.8
History of recurrent uUTI, yes	4937	4.2	11	5.3
Prior antimicrobial treatment (any), yes	70274	60	150	72
Cumulative antimicrobial prescriptions, median (IQR)	1 (0, 2)	–	<5	–
Region-level				
Quintiles of index of multiple deprivation				
Q1 (least deprived)	26869	23	42	20
Q2	23597	20	39	19
Q3	22273	19	38	18
Q4	22085	19	46	22
Q5 (most deprived)	21762	19	42	20
Rural-urban area classification				
Rural	15416	13	30	14
Urban	101170	87	177	86
Practice-level				
Practice size				
Small (<8000 patients)	30783	26	56	27
Medium (8000–11000 patients)	25377	22	44	21
Large (>11000 patients)	60426	52	107	52

Percentage values do not add up to 100% for ethnicity as these data were not available for all patients and mixed ethnicity is not shown due to limited interpretability.

A&E, accident and emergency; HgA1C, glycated haemoglobin; Q, quintile.

**Conclusions:**

Hospitalization following uUTI is rare in this study, however, at a population level, this still affects a large number of female patients. Coding limitations meant potentially linked admissions (e.g. falls and confusion) could not be identified. Hospitalizations appear to be driven by patient factors rather than regional differences or practice patterns. The role of these putative risk factors for disease progression will be explored further via multivariable logistic regression analysis.

